# Radiosynthesis and Preclinical Evaluation of Bispecific PSMA/FAP Heterodimers for Tumor Imaging

**DOI:** 10.3390/ph15030383

**Published:** 2022-03-21

**Authors:** Kongzhen Hu, Li Li, Yong Huang, Shimin Ye, Jiawei Zhong, Qingsong Yan, Yuhua Zhong, Lilan Fu, Pengju Feng, Hongsheng Li

**Affiliations:** 1 GDMPA Key Laboratory for Quality Control and Evaluation of Radiopharmaceuticals, Department of Nuclear Medicine, Nanfang Hospital, Southern Medical University, 1838 Guangzhou North Road, Guangzhou 510515, China; kzhu@smu.edu.cn (K.H.); lily145153@163.com (L.L.); shimonya@163.com (S.Y.); zhongjiawei1997@163.com (J.Z.); yan18681263235@163.com (Q.Y.); full07@163.com (L.F.); 2 National Cancer Center/National Clinical Research Center for Cancer/Cancer Hospital & Shenzhen Hospital, Chinese Academy of Medical Sciences and Peking Union Medical College, Shenzhen 518116, China; 13260455651@163.com; 3 Department of Chemistry, Jinan University, Guangzhou 510632, China; feng@163.com; 4 Department of Rehabilitation Medicine, The First School of Clinical Medicine, Southern Medical University, Guangzhou 510515, China; zhongbinyue@163.com

**Keywords:** PSMA, FAP, heterodimer, PET, tumor imaging

## Abstract

Due to tumor heterogeneity and complex tumor–stromal interactions in multicellular systems, the efficiency of monospecific tracers for tumor diagnosis and therapy is currently limited. In light of the evidence of prostate-specific membrane antigen (PSMA) overexpression in tumor cells and fibroblast activation protein (FAP) upregulation in the tumor stroma, heterodimer dual targeting PSMA and FAP may have the potential to improve tumor diagnosis. Herein, we described the radiosynthesis, in vitro characterization, and micro-PET/CT imaging of two novel ^18^F-labeled bispecific PSMA/FAP heterodimers. ^18^F-labeled heterodimers showed high specificity and affinity targeting to PSMA and FAP in vitro and in vivo. Compared with the monospecific tracers [^18^F]AlF-PSMA-BCH and [^18^F]FAPI-42, both ^18^F-labeled heterodimers exhibited better tumor uptake in tumor-bearing mice. Their favorable characterizations such as convenient synthesis, high tumor uptake, and favorable pharmacokinetic profile could lead to their future applications as bispecific radiotracers for clinical cancer imaging.

## 1. Introduction

As part of clinical diagnosis, molecular imaging is used to visualize, characterize, and measure biological processes at the cellular and molecular level using specific tracers [1]. Over the last few decades, many receptor-binding radiotracers have been explored for cancer imaging with positron emission tomography (PET), some of which have been approved for clinical use [2,3,4,5,6,7]. Compared to monomeric radiotracers, dimeric radiotracers can increase receptor numbers for targeting and increase the local ligand concentration as well as optimize pharmacokinetics. Homodimeric radiotracers have been proven to potentially improve tumor uptake and retention [8,9]. However, due to tumor heterogeneity and multicellular systems with complex tumor–stromal interactions, the typical monospecific radiotracers are of limited efficiency for diagnosis. Heterodimeric radiotracers comprise two monospecific ligands that are covalently linked with suitable linkers. Recent clinical trials have explored the use of various heterodimer radiotracers for cancer imaging and therapy [10,11,12]. The development of heterodimer radiotracers targeting multi-receptors based on biological targets could improve the efficacy of tumor targeting. In addition, the heterodimeric radiotracers could reduce patient discomfort, radiation burden, and treatment cost comparison with a combined administration of each monomeric radiotracer in the same patient.

Malignant tumors consist of neoplastic cells and the surrounding stroma, including immune cells, the extracellular matrix, and fibroblasts. Fibroblast activation protein (FAP), a type II trans-membrane serine protease, is associated with the extracellular matrix of the tumor microenvironment and is highly expressed in cancer-associated fibroblasts (CAFs), which are a subpopulation of fibroblasts in stromal cells with critical roles in tumorigenesis and metastasis [13,14,15,16]. The prostate-specific membrane antigen (PSMA) is a type II transmembrane glycoprotein overexpressed in prostate cancer epithelial cells [17,18]. Notably, PSMA is highly expressed on the neovascular endothelium of a wide variety of human solid tumors, but not on normal vascular endothelium. Co-expression of FAP and PSMA has been reported for various malignant tumors, including prostate, lung, colorectal, gastric, pancreatic, and thyroid cancers, as well as renal cell carcinomas, sarcomas, lymphomas, and other tumors [4,15,19]. Therefore, the development of heterodimers targeting dual FAP and PSMA receptors is considered a promising strategy for detecting malignant tumors.

**Figure 1:**
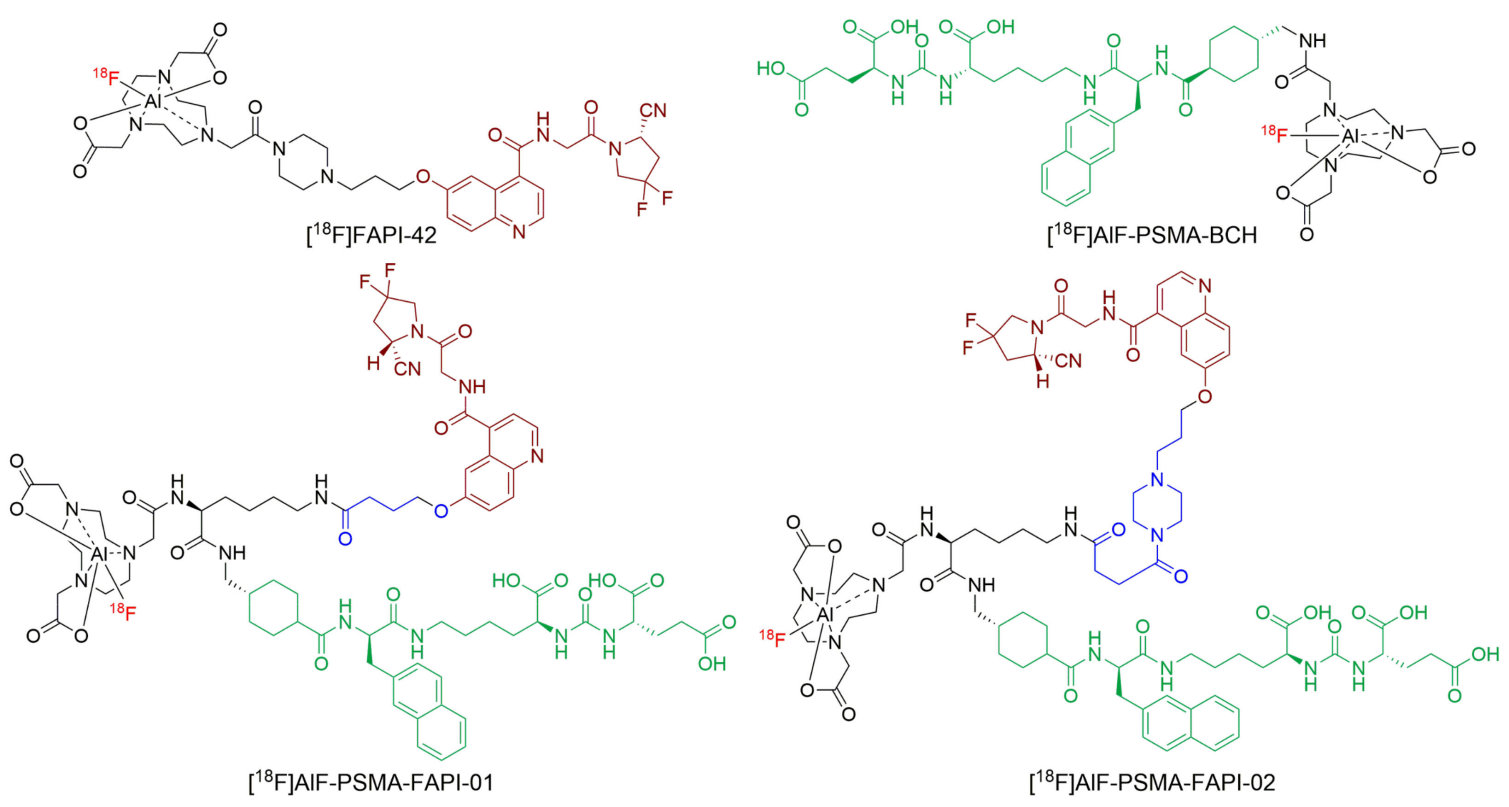
The structure of [^18^F]AlF-PSMA-FAPI-01 and [^18^F]AlF-PSMA-FAPI-02.

In this study, we report the synthesis of PSMA/FAP-targeting heterodimers that conjugated the PSMA and FAP motifs with different linkers and a 1,4,7-triazacyclononane-1,4,7-triacetic acid (NOTA)-lysine residue (Figure 1). We then labeled the heterodimer conjugate with ^18^F (*T*_1/2_ = 109.8 min, 96.7% *β*^+^, and Emean = 0.25 MeV). The ^18^F-labeled heterodimers were preclinically evaluated in terms of binding properties to PSMA and FAP, cellular characteristics, in vivo targeting, biodistribution, and imaging properties.

## 2. Results

### 2.1. Chemistry and Radiolabeling

**Scheme 1:**
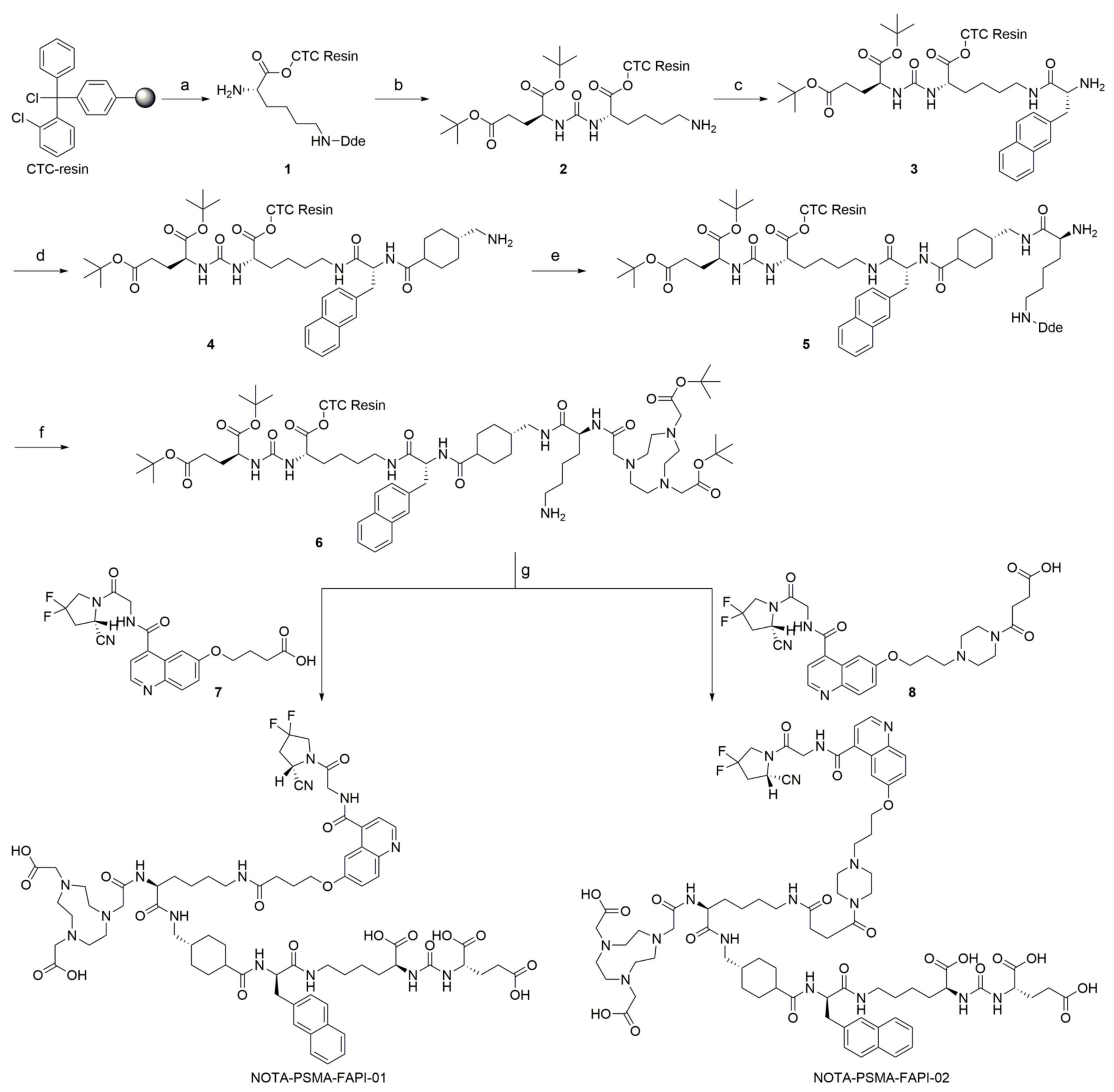
Preparation of precursors NOTA-PSMA-FAPI-01 and NOTA-PSMA-FAPI-02. Reagents and conditions: (a) (i) Fmoc-Lys(Dde)-OH, DIPEA, DCM; (ii) 20% piperidine, DMF; (b) (i) H-Glu(OtBu)_2_ HCl, DSC, DIPEA, DMF; (ii) 2% Hydrazine monohydrate, DMF; (c) (i) Fmoc-D-2-Nal-OH, DIC, HOBt, DMF; (ii) 20% piperidine, DMF; (d) (i) Fmoc-Amstat-OH, DIC, HOBt, DMF; (ii) 20% piperidine, DMF; (e) (i) Fmoc-Lys(Dde)-OH, DIC, HOBt, DMF; (ii) 20% piperidine, DMF; (f) (i) NOTA, DIC, HOBt, DMF; (ii) 2% Hydrazine monohydrate, DMF; (g) (i) compound 7 or 8, DIC, HOBt, DMF; (ii) 95% TFA, 2.5% TIPS, 2.5% H_2_O.

The bispecific PSMA/FAP-targeting heterodimers were synthesized by solid-phase peptide synthesis and then purified by RP-HPLC (Scheme 1). The heterodimers and reference compounds were obtained at >95% chemical purity and identified using mass spectrometry (Supplemental Figure S1–S4).

Radiolabeling was performed by complexation of Al^18^F in a one-step reaction. The total synthesis time was approximately 35 min and the non-decay corrected radiochemical yield was 25.7 ± 4.1% (*n* = 5) for [^18^F]AlF-PSMA-FAPI-01 and 17.5 ± 1.4% (*n* = 7) for [^18^F]AlF-PSMA-FAPI-02. The radiochemical purity was > 99% for both of ^18^F-labeled heterodimers. The molar radioactivity of [^18^F]AlF-PSMA-FAPI-01 and [^18^F]AlF-PSMA-FAPI-02 was 10–30 GBq/μmoL according to the radioactivity measurements.

### 2.2. Partition Coefficient and Stability

The *n*-octanol/PBS distribution coefficients (logD) values of [^18^F]AlF-PSMA-FAPI-01 and [^18^F]AlF-PSMA-FAPI-02 were −2.74 ± 0.02 and −2.04 ± 0.04, respectively, indicating high hydrophilicity.

**Figure 2:**
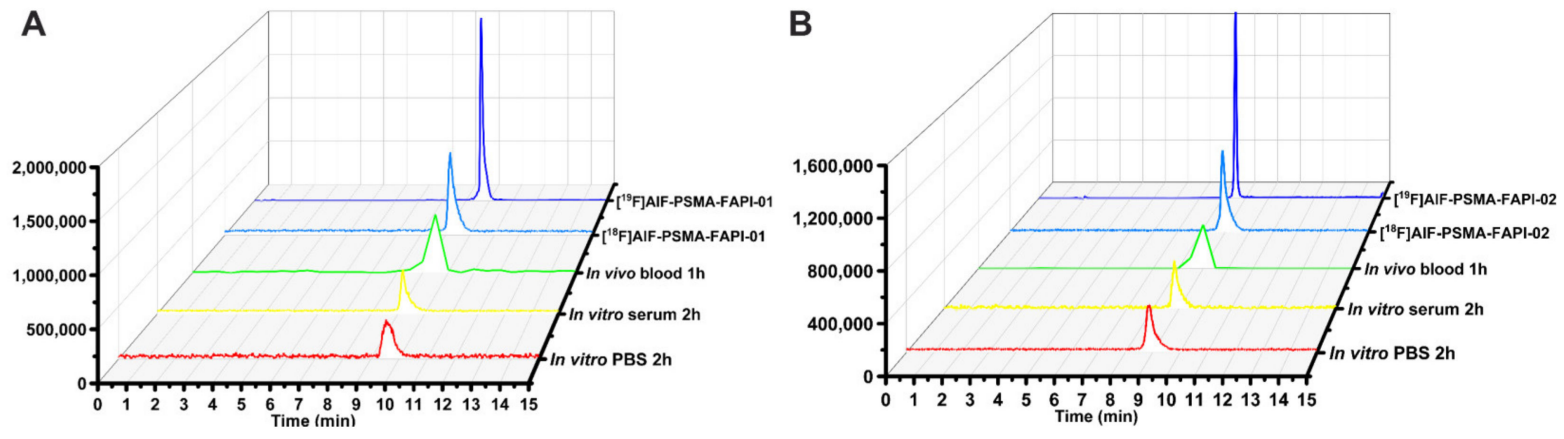
Identification and stability of [^18^F]AlF-PSMA-FAPI-01 (**A**) and [^18^F]AlF-PSMA-FAPI-02 (**B**). HPLC analysis of stability in PBS and human serum after incubation at 37 °C for 120 min and in mouse blood at 60 min p.i. The quality control radioactive chromatography of [^18^F]AlF-PSMA-FAPI-01 and [^18^F]AlF-PSMA-FAPI-02, as well as the UV chromatography of the standard [^19^F]AlF-PSMA-FAPI-01 and [^19^F]AlF-PSMA-FAPI-02.

According to the radio-HPLC results, both ^18^F-labeled heterodimers showed excellent in vitro stability in PBS (37 °C, 2 h) and in human serum (37 °C, 2 h) with no decomposition (Figure 2). We also assessed the in vivo stability of both ^18^F-labeled heterodimers in mouse blood at 1 h p.i. (Figure 2). At the end of the 1 h period, the radiochemical purity of the two radiopharmaceuticals was higher than 95% in the blood samples, indicating that both radiotracers are metabolically stable in vivo. Overall, both ^18^F-labeled heterodimers [^18^F]AlF-PSMA-FAPI-01 and [^18^F]AlF-PSMA-FAPI-02 exhibited excellent stability in vitro and in vivo within the time tested.

### 2.3. Cell Studies

**Figure 3:**
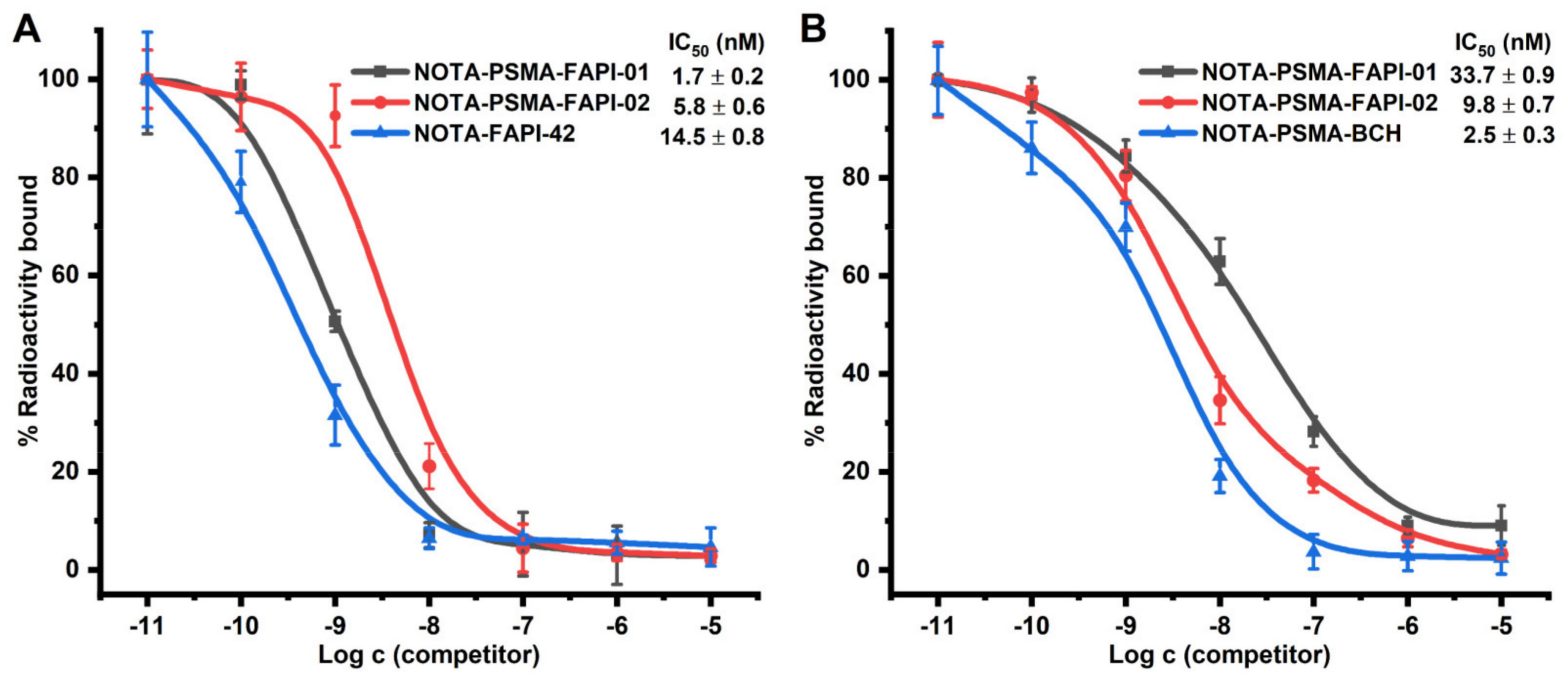
(**A**) Competitive binding of the heterodimers NOTA-PSMA-FAPI-01 and NOTA-PSMA-FAPI-02 and the corresponding monomer NOTA-FAPI-42 to FAP on A549-FAP cells (*n* = 6). (**B**) Competitive binding of the heterodimers NOTA-PSMA-FAPI-01 and NOTA-PSMA-FAPI-02 and the corresponding monomer NOTA-PSMA-BCH to PSMA on 22Rv1 cells (*n* = 6).

We performed competition binding experiments to determine the binding potential. Different concentrations of unlabeled precursor (10^−5^–10^−11^ M) were added to cells with a fixed activity of radiolabeled. The results are expressed as IC_50_ values of the two heterodimers compared to the monospecific references NOTA-FAPI-42 and NOTA-PSMA-BCH. In FAP-expressing A549-FAP cells, NOTA-PSMA-FAPI-01 (IC_50_ = 1.7 nM) and NOTA-PSMA-FAPI-02 (IC_50_ = 5.8 nM) were approximately 9- and 3-fold higher in potency, respectively, than the monomer NOTA-FAPI-42 (IC_50_ = 14.5 nM; Figure 3A). In contrast, NOTA-PSMA-FAPI-01 (IC_50_ = 33.7 nM) and NOTA-PSMA-FAPI-02 (IC_50_ = 9.8 nM) were approximately 13- and 4-fold less potent, respectively, than the monomer NOTA-PSMA-BCH (IC_50_ = 2.5 nM) in PSMA-expressing 22Rv1 cells (Figure 3B). The results of these two sets of experiments indicate that both heterodimers possess potent dual PSMA and FAP receptor-binding affinities with nanomolar IC_50_ values.

**Figure 4:**
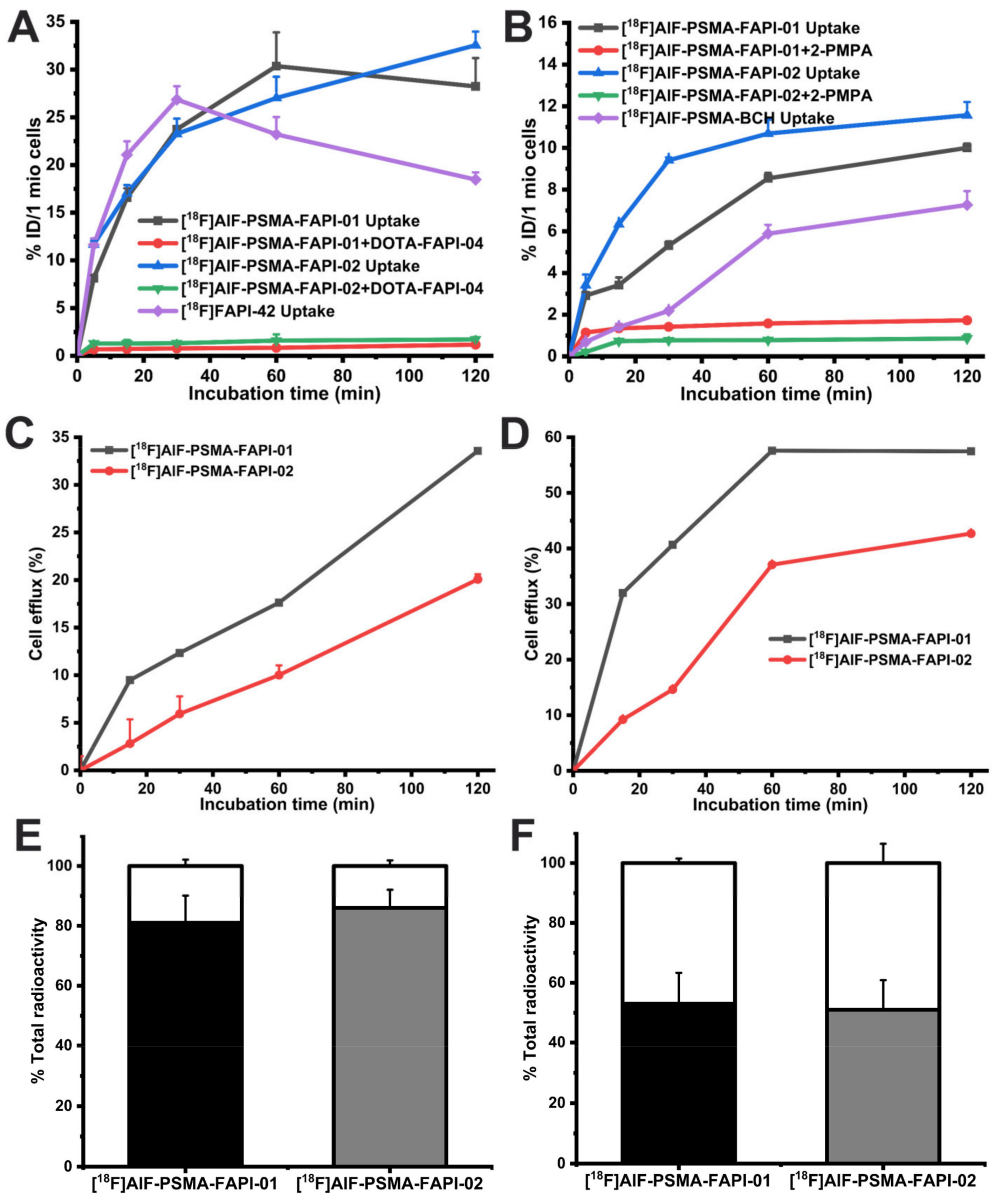
In vitro cell uptake and blocking study results of [^18^F]AlF-PSMA-FAPI-01, [^18^F]AlF-PSMA-FAPI-02, and corresponding monomeric tracer [^18^F]FAPI-42 or [^18^F]AlF-PSMA-BCH in A549-FAP cells (**A**) and 22Rv1 cells (**B**). Efflux kinetics of [^18^F]AlF-PSMA-FAPI-01 and [^18^F]AlF-PSMA-FAPI-02 after 60 min incubation of A549-FAP cells (**C**) and 22Rv1 cells (**D**) with tracers followed by incubation with compound-free medium for 15–120 min. Values indicate the mean ± SD (*n =* 6). Internalized and membrane-bound [^18^F]AlF-PSMA-FAPI-01 and [^18^F]AlF-PSMA-FAPI-02 in A549-FAP (**E**) and 22Rv1 cells (**F**) after incubation for 60 min. The black and grey segments represent the internalized proportions, while the blank segments indicate the membrane-bound fraction.

Next, to investigate the binding specificity of [^18^F]AlF-PSMA-FAPI-01 and [^18^F]AlF-PSMA-FAPI-02 to FAP and PSMA, cell uptake and blocking studies were performed with A549-FAP and 22Rv1 cells (Figure 4A). The cell uptake radioactivity levels of both ^18^F-labeled heterodimers on A549-FAP and 22Rv1 cells showed similar binding kinetics increases in a time-dependent manner and were higher than that monomer [^18^F]FAPI-42 or [^18^F]AlF-PSMA-BCH. Both [^18^F]AlF-PSMA-FAPI-01 and [^18^F]AlF-PSMA-FAPI-02 showed higher cell uptake in A549-FAP cells than in 22Rv1 cells, which may be due to the higher density of FAP receptors in A549-FAP cells relative to the density of PSMA receptors in 22Rv1 cells. Additionally, the uptake of both heterodimers on A549-FAP and 22Rv1 cells was significantly reduced after incubation with an excess of the corresponding blocking substance (Supplemental Table S1). The results showed that both ^18^F-labeled heterodimers can specifically bind to dual FAP and PSMA receptors.

The data from the cell-based internalization assays demonstrated that both ^18^F-labeled heterodimers had high internalization in A549-FAP cells with values of more than 80% and moderate internalization in 22Rv1 cells with values of approximately 50% after 60 min incubation (Figure 4C). The efflux experiments showed that both ^18^F-labeled heterodimers had low cell efflux rates in A549-FAP cells and moderate cell efflux rates 22Rv1 cells after 2 h incubation (Figure 4B). Compared to [^18^F]AlF-PSMA-FAPI-01, [^18^F]AlF-PSMA-FAPI-02 exhibited lower cellular efflux in both A549-FAP and 22Rv1 cells during the 2 h incubation time.

### 2.4. Tissue Biodistribution Studies

**Figure 5:**
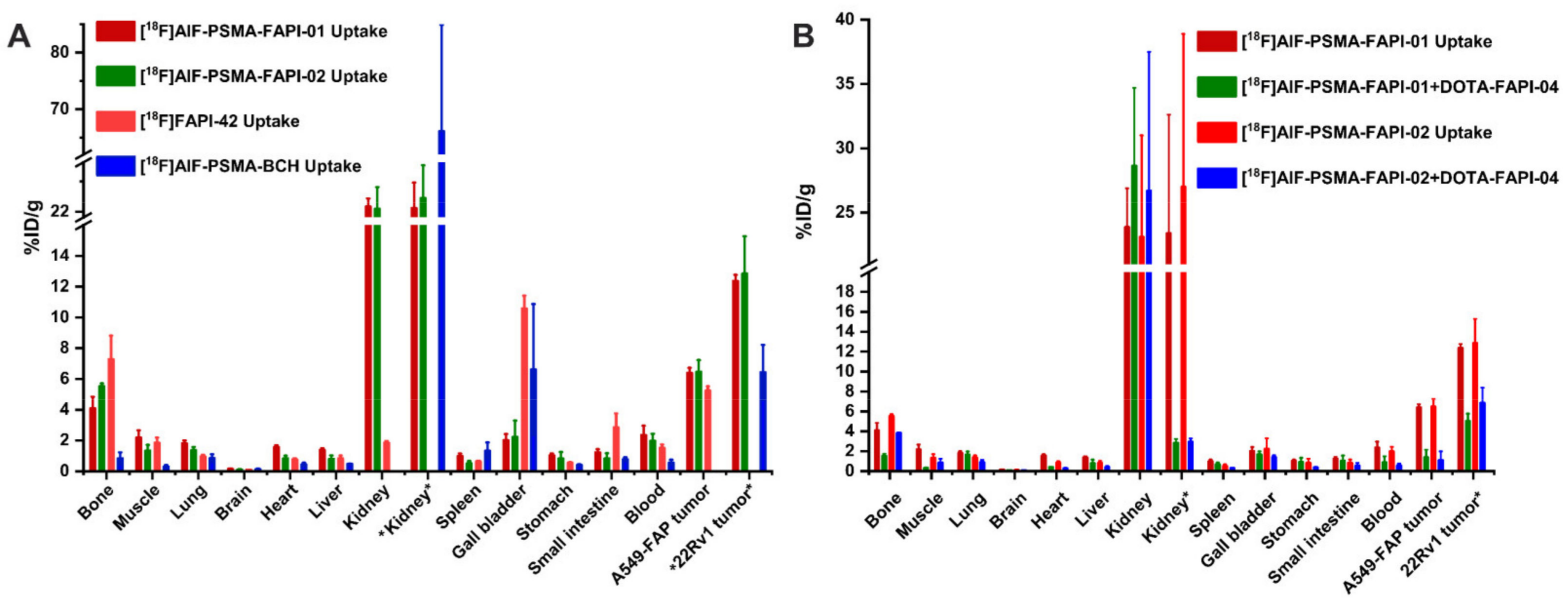
(**A**) Biodistribution of heterodimeric tracers [^18^F]AlF-PSMA-FAPI-01 and [^18^F]AlF-PSMA-FAPI-02 in A549-FAP or 22Rv1 tumor-bearing nude mice at 60 min p.i.,which compared with the corresponding monomer [^18^F]FAPI-42 in A549-FAP tumor-bearing mice or [^18^F]AlF-PSMA-BCH in 22Rv1 tumor-bearing mice. (**B**) Blocking experiments of [^18^F]AlF-PSMA-FAPI-01 and [^18^F]AlF-PSMA-FAPI-02 in A549-FAP xenografts with coinjection of DOTA-FAPI-04 inhibitor (3 mg/kg), while the kidney and 22Rv1 tumor labeled “*” are from 22Rv1 tumor-bearing mice using 2-PMPA competitor (3 mg/kg). All the data are expressed as mean ± SD (n = 4 in each group).

To assess the bispecific receptors′ binding abilities in vivo, biodistribution and blocking experiments were performed 1 h p.i., as shown in Figure 5 and Supplemental Table S2. For blocking, 3 mg/kg of DOTA-FAPI-04 was co-injected in A549-FAP xenografts or 3 mg/kg 2-PMPA competitor in 22Rv1 xenografts. Both heterodimeric tracers showed higher tumor uptake in A549-FAP and 22Rv1 tumor-bearing mice compared to the corresponding monomeric tracers (6.40 ± 0.32 %ID/g for [^18^F]AlF-PSMA-FAPI-01 and 6.47 ± 0.76 %ID/g [^18^F]AlF-PSMA-FAPI-02 vs. 5.25 ± 0.27 %ID/g for [^18^F]FAPI-42 in A549-FAP tumors, *p* < 0.05; 12.37 ± 0.40 %ID/g for [^18^F]AlF-PSMA-FAPI-01 and 12.85 ± 2.43 %ID/g for [^18^F]AlF-PSMA-FAPI-02 vs. 6.44 ± 1.78 %ID/g for [^18^F]PSMA-BCH in 22Rv1 tumors, *p* < 0.05) (Figure 5A and Supplemental Table S2). Radioactivity uptake in A549-FAP and 22Rv1 tumors was significantly decreased when co-injected with the corresponding competitors (6.40 ± 0.32 %ID/g vs. 1.38 ± 0.77 %ID/g for [^18^F]AlF-PSMA-FAPI-01 and 6.47 ± 0.76 %ID/g vs. 1.11 ± 0.88 %ID/g [^18^F]AlF-PSMA-FAPI-02 in A549-FAP tumors, *p* < 0.05; 12.37 ± 0.40 %ID/g vs. 5.03 ± 0.75 %ID/g for [^18^F]AlF-PSMA-FAPI-01 and 12.85 ± 2.43 %ID/g vs. 6.85 ± 1.53 %ID/g for [^18^F]AlF-PSMA-FAPI-02 in 22Rv1 tumors, *p* < 0.05) (Figure 5B). Of note, the ^18^F-labeled heterodimeric tracers showed high uptake in bone and could be blocked by the FAP competitor DOTA-FAPI-04 (4.09 ± 0.76 %ID/g vs. 1.53 ± 0.21 %ID/g for [^18^F]AlF-PSMA-FAPI-01 and 5.53 ± 0.18 %ID/g vs. 3.83 ± 0.02 %ID/g [^18^F]AlF-PSMA-FAPI-02, *p* < 0.05), suggesting specific FAP binding to bone marrow (Figure 5A,B). Similarly, the tracers showed high uptake in kidneys, which were significantly decreased by the PSMA competitor 2-PMPA (23.4 ± 9.22 %ID/g vs. 2.85 ± 0.38 %ID/g for [^18^F]AlF-PSMA-FAPI-01 and 27.0 ± 11.9 %ID/g vs. 2.94 ± 0.35 %ID/g [^18^F]AlF-PSMA-FAPI-02, *p* < 0.001), suggesting specific PSMA binding to kidneys (Figure 5A,B).

### 2.5. Micro-PET Imaging

**Figure 6:**
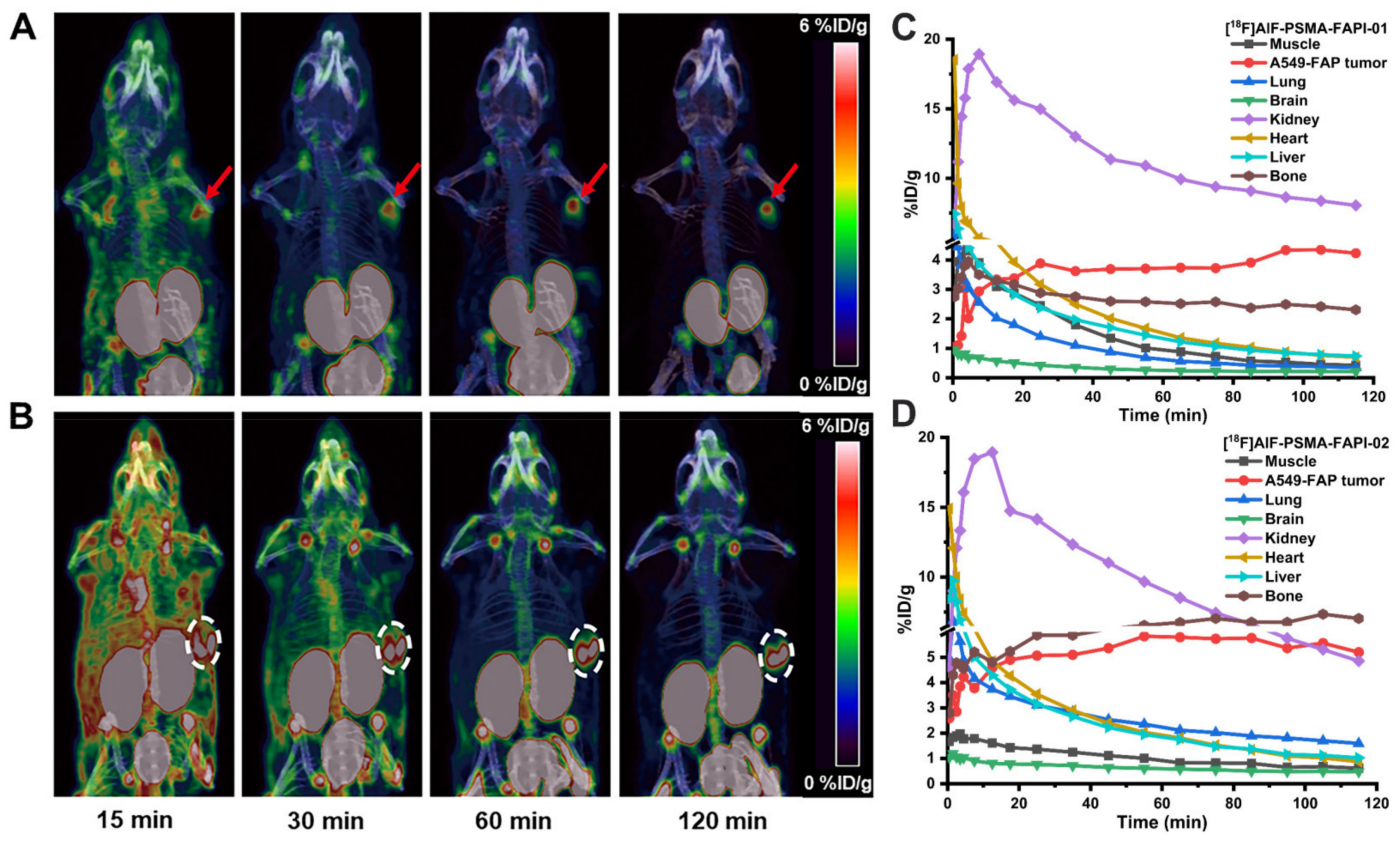
Maximum intensity projection (MIP) 15, 30, 60, and 120 min p.i. of [^18^F]AlF-PSMA-FAPI-01 (**A**) and [^18^F]AlF-PSMA-FAPI-02 (**B**) in A549-FAP tumor-bearing mice. Arrows and circles represent the locations of the A549-FAP tumors. Time-activity curves of [^18^F]AlF-PSMA-FAPI-01 (**C**) and [^18^F]AlF-PSMA-FAPI-02 (**D**) in selected organs up to 120 min p.i.

Dynamic micro-PET studies were conducted using the ^18^F-labeled heterodimeric tracers [^18^F]AlF-PSMA-FAPI-01 and [^18^F]AlF-PSMA-FAPI-02 and the corresponding monomeric tracers [^18^F]FAPI-42 and [^18^F]AlF-PSMA-BCH in nude mice bearing A549-FAP or 22Rv1 tumor xenografts. The representative maximum intensity projection (MIP) images at different times and time–activity curves of [^18^F]AlF-PSMA-FAPI-01 and [^18^F]AlF-PSMA-FAPI-02 over the course of 2 h p.i. revealed fast biodistribution and specific uptake in nude mice bearing A549-FAP tumor xenografts in vivo (Figure 6). The A549-FAP tumors were visible soon after injection (15 min p.i.) and showed constantly improving contrast over the duration of the PET scan with both heterodimeric tracers (Figure 6A,B). The time–activity curves of [^18^F]AlF-PSMA-FAPI-01 and [^18^F]AlF-PSMA-FAPI-02 demonstrated rapid uptake and excellent retention in tumors, as well as rapid clearance from non-target organs via the kidneys (Figure 6C,D). Brain, muscle, lung, heart, and liver were all low for both [^18^F]AlF-PSMA-FAPI-01 and [^18^F]AlF-PSMA-FAPI-02.

**Figure 7:**
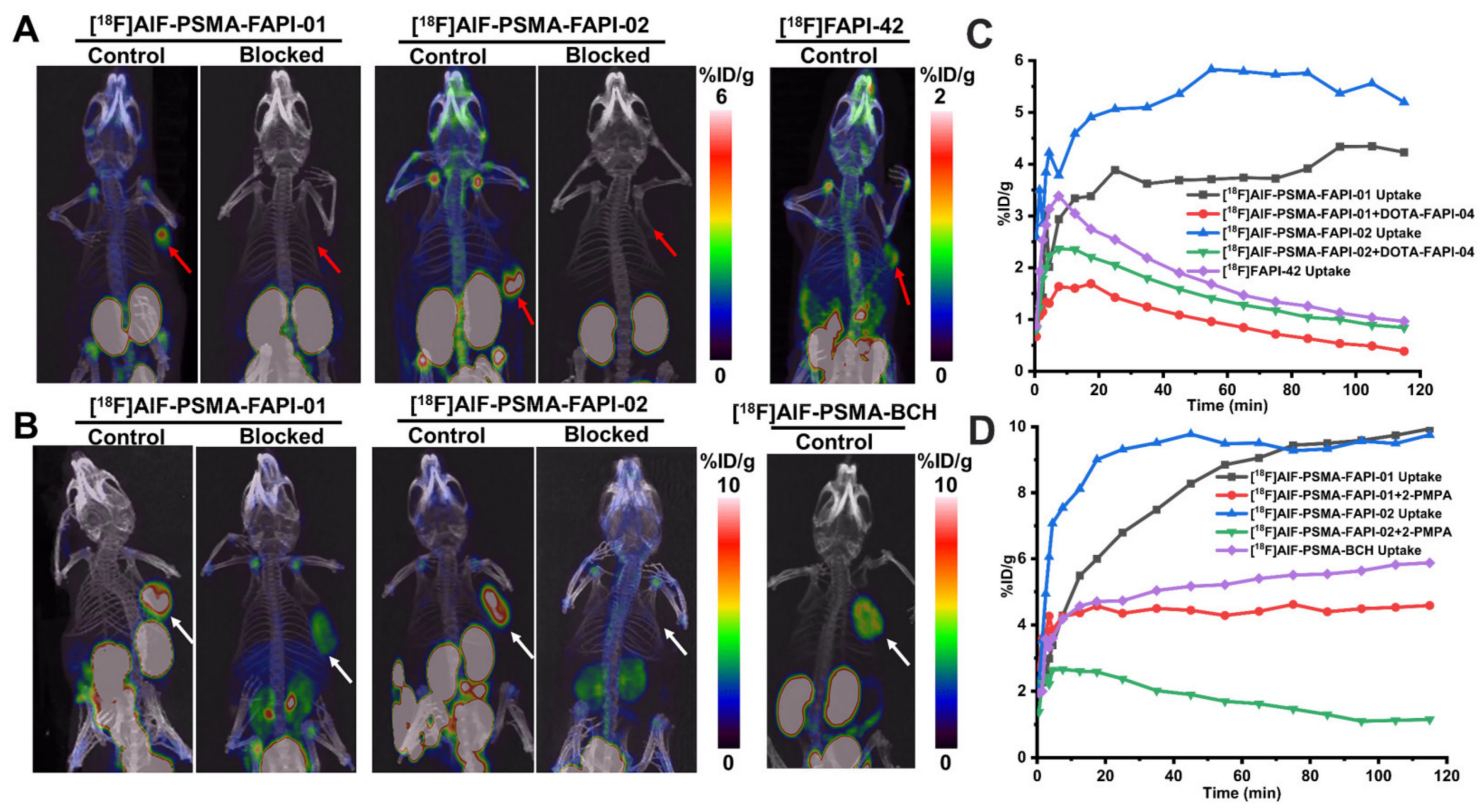
(**A**) Representative MIP images of A549-FAP xenografts at 120 min p.i. of [^18^F]AlF-PSMA-FAPI-01, [^18^F]AlF-PSMA-FAPI-02, and [^18^F]FAPI-42 with and without the FAP competitor DOTA-FAPI-04 (3 mg/kg). The A549-FAP tumors are indicated by red arrows. (**B**) Representative MIP images of 22Rv1 xenografts at 120 min p.i. of radiotracers with and without the PSMA competitor 2-PMPA (3 mg/kg). The 22Rv1 tumors are indicated by white arrows. Time–activity curves of A549-FAP tumors (**C**) and 22Rv1 tumors (**D**) up to 120 min p.i.

Next, a comparison of tumor uptake and blocking experiments of [^18^F]AlF-PSMA-FAPI-01 and [^18^F]AlF-PSMA-FAPI-02 and the corresponding monomers [^18^F]FAPI-42 and [^18^F]AlF-PSMA-BCH were performed with mice bearing 22Rv1 or A549-FAP tumors (Figure 7). Consistent with the biodistribution data, tumor uptake of the heterodimeric tracers [^18^F]AlF-PSMA-FAPI-01 and [^18^F]AlF-PSMA-FAPI-02 was higher than that of the monomeric tracers [^18^F]FAPI-42 and [^18^F]AlF-PSMA-BCH in both 22Rv1 and A549-FAP tumors 20 min p.i. When co-injected with the respective competitors, the tumor uptake of both [^18^F]AlF-PSMA-FAPI-01 and [^18^F]AlF-PSMA-FAPI-02 in A549-FAP and 22Rv1 tumors significantly decreased, implying dual targeting FAP/PSMA specificity in vivo. In the kidneys, a strong tendency of decreased radioactivity uptake was observed for the PSMA-blocked group; this result was expected due to the endogenous expression of PSMA in kidneys [20,21,22]. There was also apparent specific uptake of [^18^F]AlF-PSMA-FAPI-01 and [^18^F]AlF-PSMA-FAPI-02 in the shoulder joints and knee joints, which was consistent with previous reports of the FAP tracers [^18^F]FAPI-42, [^18^F]FGlc-FAPI, and [^18^F]AlF-P-FAPI [23,24,25].

## 3. Discussion

PSMA and FAP are two abundantly expressed biological targets on cancer. Several PSMA and FAP radiotracers have been developed and successfully investigated in human clinical trials [3,4,5,26,27,28]. However, the application of monomeric receptor-based radiotracers is limited by changes in receptor expression due to tumor heterogeneity, binding affinity, and suboptimal in vivo pharmacokinetics. To take advantage of the favorable properties of heterodimers, we designed and synthesized bispecific heterodimer radiotracers targeting both PSMA and FAP for cancer imaging.

Based on the lysine-ureido-glutamate pharmacophore for PSMA targeting and quinoline-based pharmacophore for FAP targeting, the two moieties were conjugated via a linker and a NOTA chelator, which is ideal for ^18^F complexation [^18^F]Fluoride aluminum [23,26]. In a previous study, we demonstrated that the linkers can affect metabolic stability and tumor uptake in vivo [23]. Thus, we introduced the changed linkers to modulate the molecular weight and pharmacokinetics of the heterodimers.

As a dual functional radiotracer, the heterodimer should specifically bind to each receptor. The results of the competitive binding assay demonstrated that both heterodimers can potently bind FAP and PSMA in vitro. Moreover, the dual-receptor specificity of the heterodimer tracers was confirmed by the blocking studies. Both heterodimeric tracers showed higher internalization in the FAP-positive A549-FAP cells than that in the PSMA-positive 22Rv1 cells, which is consistent with the internalization findings for previously reported monomeric tracers [20,21,23,25,29,30,31,32]. The distinct characteristics of the heterodimeric tracers′ internalization and efflux in the cells also contributed to the higher uptake in A549-FAP cells compared to 22Rv1 cells.

The comparative biodistribution experiments revealed that the heterodimeric tracers combining PSMA and FAP pharmacophores retained the characteristics of their monomers related to uptake in tumors and peripheral organs expressing PSMA and FAP, such as 22Rv1 tumors (PSMA), kidneys (PSMA), A549-FAP tumors (FAP), and bone (FAP). Furthermore, the blocking studies with 2-PMPA and DOTA-FAPI-04 showed significantly diminished uptake in tumors and the “in vivo reference” organs, demonstrating their specific binding to these receptors.

As detailed in the recent literature, one of the main drawbacks of ^18^F-labeled FAP radiotracers including [^18^F]FAPI-42, [^18^F]FGlc-FAPI, and [^18^F]AlF-P-FAPI, is their unfavorable hepatobiliary excretion with high intestinal or gallbladder uptake in animal models [23,24,25]. In the present study, the high uptake and rapid washout in the kidney reflected both the expected renal clearance of the radiotracers and the relatively high expression of PSMA in murine kidneys [20,21,22], as evidenced by the dynamic time–activity curves and blocking experiment with 2-PMPA. The altered kinetics of heterodimeric tracers compared with the monomer [^18^F]FAPI-42 may be attributed to differences in hydrophilicity, molecular size and charge, and metabolic stability. In addition, tumor uptake for [^18^F]AlF-PSMA-FAPI-01 and [^18^F]AlF-PSMA-FAPI-02 was higher than that for the corresponding monomers [^18^F]FAPI-42 and [^18^F]AlF-PSMA-BCH in FAP-positive A549-FAP and PSMA-positive 22Rv1 tumor xenografts. The limitation of this study is the mouse models from cancer cell-derived xenografts (CDXs), which cannot express both FAP and PSMA receptors in one tumor-bearing model. Both ^18^F-labeled heterodimeric radiotracers will be further performed and compared by the prostate cancer patient-derived xenografts (PDXs), which established by direct implantation of fresh surgical tissue fragments into immunodeficient mice, could display the “synergistic effect” of heterodimers because they retain the tumor microenvironment and molecular signatures of the parental prostate cancer compared to that of CDXs. Overall, these results are consistent with the hypothesis that the use of heterodimers can improve tumor targeting efficacy and optimize pharmacokinetics.

The heterodimer strategy is often a double-edged sword for developing radiopharmaceuticals. It may improve the efficacy of tumor detection but may also increase the risk of false-positive results. The development of heterodimer radiotracers may provide us a window to optimize the radiotracer structure for tumor diagnosis with avoiding a combined administration of each monomeric radiotracer.

## 4. Materials and Methods

### 4.1. Chemistry

All reagents were commercially purchased and used without further purification unless otherwise indicated. NOTA-FAPI-42 and NOTA-PSMA-BCH were purchased from Nanchang TanzhenBio Co., Ltd. (Nanchang, China) and had high chemical purity (> 95%). Further details of the synthesis method of NOTA-PSMA-FAPI-01 and NOTA-PSMA-FAPI-02, as well as the corresponding reference standards [^19^F]AlF-PSMA-FAPI-01 and [^19^F]AlF-PSMA-FAPI-02, are provided in the Supplemental Materials.

### 4.2. Radiosynthesis

[^18^F]Fluoride was produced by irradiation of a high pressure [^18^ O]H_2_O target with 18 MeV proton beams using a PET trace biomedical cyclotron (PET 800, General Electric, Boston, MA, USA). Radioactivity was measured using a Capintec CAPRAC-R dose calibrator (NJ, USA). Radiosynthesis was carried out manually as previously described [20,23,26]. In brief, [^18^F]F^−^ (3.7–7.4 GBq) was trapped on a Sep-Pak Plus QMA (Waters Corporation, No:034339115A, preconditioned with 5 mL of 0.5 M sodium acetate buffer pH 3.9 and 10 mL of water) and eluted using 0.30 mL of 0.5 M sodium acetate buffer pH 3.9 to a mixture of AlCl_3_ (40.0 nmoL, 20.0 μL, and 2.0 mM in 0.2 M sodium acetate buffer pH 4.0) and NOTA precursor (80.0 nmol) in 300 μL dimethyl sulfoxide (DMSO). The mixture was then heated for 15 min at 100 °C before being cooled, diluted with 5 mL of water, and transferred over an activated C18 cartridge (Waters Corporation, No: 04330161A, preconditioned with 5 mL ethanol and 10 mL water). Next, the C18 cartridge was washed with 20 mL of water and eluted with 1 mL of ethanol/water (1/1, *v*/*v*). The elution was evaporated with nitrogen flow at 40 °C for 10 min. Lastly, the dried product was formulated in normal saline and passed through a sterile Millipore filter (Millipore, No: R0PB47001, 0.22 μm) into a sterile vial and stored until further use. The molar activity of ^18^F-labeled heterodimers is described in the Supplemental Materials.

### 4.3. Partition Coefficient

An aliquot (74 kBq) of [^18^F]AlF-PSMA-FAPI-01 or [^18^F]AlF-PSMA-FAPI-02 was added to a centrifuge tube containing a mixture of phosphate-buffered solution (PBS, pH 7.4, 5.0 mL) and *n*-octanol (5.0 mL). The mixture was vigorously vortexed at ambient temperature for 5 min and then centrifuged (10,000 rpm, 5 min). Three samples (each 100 µL) from each phase underwent radioactivity measurement using a γ-counter (CAPRAC-R, Capintec, Inc., Ramsey, NJ, USA).

### 4.4. In Vitro Serum Stability

An aliquot (3.7 MBq) of [^18^F]AlF-PSMA-FAPI-01 or [^18^F]AlF-PSMA-FAPI-02 was added to PBS (200 μL) or human serum (200 μL) and incubated for 2 h at 37 °C. For the PBS study, an aliquot of the solution was directly injected into a radio high-performance liquid chromatography (radio-HPLC) for analysis. For the human serum study, an equal volume of acetonitrile (200 μL) was added to the mixture to precipitate the plasma proteins. The mixture was centrifuged (rpm, 5 min) and the supernatant was then analyzed by radio-HPLC.

### 4.5. In Vivo Stability

All animal experiments were performed in compliance with the Nanfang Hospital Animal Ethics Committee at the Southern Medical University (Application No: NFYY-2020-189). The in vivo stability was assessed as previously described [20,23]. Kunming mice were sacrificed 60 min after intravenous injection of the radiotracer (0.37–0.74 GBq/kg per mouse). Blood samples (400 µL) were then collected and added to an equal volume of acetonitrile. After centrifugation (10,000 rpm, 5 min), the radioactivity of the supernatant and precipitate was measured using a γ-counter. A sample of the supernatant (100 µL) was used for subsequent HPLC analysis. The eluted samples were manually collected within 15 min at intervals of 30 s and measured using a γ-counter. The counts of the samples were plotted as intensity (cpm) versus fractions.

### 4.6. Cell Culture

PSMA-positive 22Rv1 cells (purchased from Institute of Biochemistry and Cell Biology, Shanghai, China) and stably FAP-transfected A549-FAP cells (acquired using lentiviral infection) were used for the cell-based experiments. The 22Rv1 cells were cultivated in RPMI-1640 medium (Gibco) at 37 °C/5% carbon dioxide, while the A549-FAP cells were cultivated in DMEM medium. Both media were supplemented with 10% heat-inactivated fetal bovine serum (FBS) and 1% penicillin/streptomycin (Gibco). Puromycin (1 µg/mL) was also added to the media to maintain FAP expression.

### 4.7. In Vitro Cell Study

Cells were seeded in 12-well plates 2 days in advance to achieve a final confluence of approximately 80–90% (4–5 × 10^5^ cells/well). The medium was displaced by 1 mL fresh medium without FBS. The method of cell studies was similar to a method reported previously [31]. For the competitive binding experiments, cells were incubated with the known radiotracers ([^18^F]FAPI-42 for the FAP receptor and [^18^F]AlF-PSMA-BCH for PSMA) in the presence of 7 different concentrations (10^−5^–10^−11^ M) of the corresponding unlabeled heterodimers or corresponding monomer and incubated for 60 min at 37 °C. In all experiments, the cells were washed twice with PBS, lysed with 1 M NaOH, and counted using a γ-counter. IC_50_ values were calculated by fitting the data using a nonlinear regression algorithm (implemented in SPSS). For the radioligand binding studies, the cells were incubated for 5, 15, 30, 60, or 120 min at 37 °C after the addition of the heterodimer or corresponding monomeric radiotracer radiotracers. The blocking experiments were determined by adding the corresponding competitors (4.4 μM 2-PMPA for 22Rv1 cells and 2.3 μM DOTA-FAPI-04 for A549-FAP cells). Radioactivity was measured using a γ-counter, normalized to 1 million cells (1 mio cells), and calculated as the percentage of the applied dose (%ID/1 mio cells). For the internalization experiments, the cells were incubated with the radiolabeled compound for 60 min, washed twice with 1 mL PBS, and then incubated for 10 min with 1 mL glycine HCl (1 M, pH 2.2) to detach the extracellular bound tracer. Next, the cells were washed with PBS (2.0 mL) and lysed with 1 M NaOH (1.5 mL). The radioactivity of the acid wash solution (extracellularly bound tracer) and cell lysate (internalized tracer) was assessed separately using a γ-counter. For the efflux experiments, the radioactive medium was removed after 60 min incubation and replaced with non-radioactive medium. At predetermined time points of 15, 30, 60, and 120 min, the cells were washed with 1.0 mL PBS three times and then lysed. The radioactivity of the remaining cells was assessed using a γ-counter. Each experiment was conducted three times with three or four replicates per independent experiment.

### 4.8. Tumor Xenotransplantation

For the biodistribution and micro-PET imaging studies, male BALB/c nude mice (6–8 weeks) were subcutaneously inoculated with 1 × 10^7^ 22Rv1 or 5 × 10^6^ A549-FAP cells in the right shoulder. Mice were imaged or used in the biodistribution studies when the tumor reached 5–10 mm in diameter.

### 4.9. Tissue Biodistribution Studies

Mice bearing 22Rv1 or A549-FAP (*n* = 4 in each group) tumors were euthanized 60 min after intravenous administration of each tracer (740–1480 KBq/mouse). In the blocking group, the same experiment was conducted by co-injecting 50 μg of 2-PMPA for mice bearing 22Rv1 tumors or 50 μg of DOTA-FAPI-04 for mice bearing A549-FAP tumors. The organs of interest and tumors were quickly dissected and weighed. Radioactivity was assessed using a γ-counter and calculated as the percentage injected dose per gram (%ID/g).

### 4.10. Micro-PET Imaging

Mice bearing xenografted 22Rv1 or A549FAP tumors (*n* = 3 per group) were anesthetized and placed in the prone position in a micro-PET scanner (Siemens, Erlangen, Germany). Dynamic PET/CT images were acquired 2 h post-injection (p.i.) with the radiotracer (5.55–11.1 MBq). The blocking experiments were performed by co-injecting 3 mg/kg of 2-PMPA for mice bearing 22Rv1 tumors or 3 mg/kg of DOTA-FAPI-04 for mice bearing A549-FAP tumors. The images were reconstructed using a three-dimensional ordered-subset expectation maximum algorithm and converted to %ID/g. Attenuation correction was performed using the unenhanced low-dose CT data. For data analysis, the regions of interest (ROIs) were manually drawn over the tumors and major organs on decay-corrected whole-body coronal images obtained using Inevon Research Workplace 4.1 software.

### 4.11. Statistical Analysis

Data were expressed as the mean ± standard deviation (SD). The significance of comparisons between two datasets was determined using statistical tests performed with SPSS 22.0 software (IBM Corp., Armonk, NY, USA). *p*-values < 0.05 were considered statistically significant.

## 5. Conclusions

Two novel ^18^F-labeled PSMA-FAP heterodimeric radiotracers containing both PSMA and FAP pharmacophores were designed and synthesized for dual PSMA and FAP-targeted tumor imaging. Both ^18^F-labeled heterodimeric radiotracers exhibited dual PSMA and FAP targeting properties in vitro and in vivo. Compared with the monomers, the dual-targeting heterodimeric radiotracers showed increased tumor uptake and retention in PSMA and FAP-positive tumors. Taken together, these findings suggest that ^18^F-labeled heterodimeric radiotracers with high affinity, specificity, high tumor uptake, and favorable pharmacokinetic profile are promising bispecific radiotracers for noninvasive imaging of tumors with both or either receptor expression pattern.

## Data Availability

Data is contained within the article or Supplementary Materials.

## Supplementary Materials

The following supporting information can be downloaded online at: https://www.mdpi.com/article/10.3390/ph15030383/s1, The syntheses of the precursors NOTA-PSMA-FAPI-01 and NOTA-PSMA-FAPI-02, as well as the corresponding reference standards [^19^F]AlF-PSMA-FAPI-01 and [^19^F]AlF-PSMA-FAPI-02; Mass spectrometry Data; Molar activity; Cell uptake and blocking data; Biodistribution Table.

## Abbreviations

CAFs: cancer-associated fibroblasts; DMSO, dimethyl sulfoxide; FAP, fibroblast activation protein; % ID/g, percentage of injected dose per gram of tissue; MIP, maximum intensity projections; mio cells, million cells; NOTA, 1,4,7-triazacyclononane-1,4,7-triacetic acid; PBS, phosphate buffer solution; PET, positron emission tomography; PSMA, prostate specific membrane antigen; radio-HPLC, radio-high performance liquid chromatography; ROIs, regions of interest; SD, standard deviation.
