# Supplementary material for: Radiosynthesis and Preclinical Evaluation of Bispecific PSMA/FAP Heterodimers for Tumor Imaging
Source: Pharmaceuticals (Basel). 2022 Mar 21;15(3):383. doi: 10.3390/ph15030383 (PMC8949503; doi:10.3390/ph15030383)
Supplement: Supplementary file 1 [file pharmaceuticals-15-00383-s001.zip › pharmaceuticals-1587481-supplementary.pdf]

Supplementary Material

Radiosynthesis and preclinical evaluation of bispecific PSMA/FAP heterodimers for tumor imaging

Running title Bispecific heterodimers for tumor imaging

Kongzhen Hu [a,1], Li Li [a,1], Yong Huang [b], Shimin Ye [a], Jiawei Zhong [c], Qingsong Yan [c], Yuhua Zhong [d], Lilan Fu [a], Pengju Feng [c], Hongsheng Li [a,*]

[a] *GDMPA Key Laboratory for Quality Control and Evaluation of Radiopharmaceuticals, Department of Nuclear Medicine, Nanfang Hospital, Southern Medical University,1838 Guangzhou North Road, Guangzhou, Guangdong Province, 510515, China*

[b]*National Cancer Center/National Clinical Research Center for Cancer/Cancer Hospital & Shenzhen Hospital, Chinese Academy of Medical Sciences and Peking Union Medical College, Shenzhen 518116, China*

[c]*Department of Chemistry, Jinan University, Guangzhou 510632, China*

[d]*Department of Rehabilitation Medicine, The First School of Clinical Medicine, Southern Medical University, Guangzhou, Guangdong Province 510515, China*

[*]Corresponding author. Department of Nuclear Medicine, Nanfang Hospital, Southern Medical University, Guangzhou 510515, China

Email address: lhs0425@126.com (Hongsheng Li)

[1] Kongzhen Hu and Li Li contributed equally to this work.

Table of Contents

Chemistry 3

Synthesis of heterodimeric compound NOTA-PSMA-FAPI-01 3

Synthesis of heterodimeric compound NOTA-PSMA-FAPI-02 5

Synthesis of reference compound [^{19}F]AlF-PSMA-FAPI-01/02 6

Molar activity..8

Cell uptake and blocking study…………………………………………...9

Biodistribution and blocking study……………………………………...10

Chemistry

Synthesis of heterodimeric compound NOTA-PSMA-FAPI-01

CTC Resin (471 mg, 0.5 mmol, 1.0 equiv) was swelling in 20.0 mL CH_2Cl_2 (DCM) and was added Fmoc-Lys(Dde)-OH (533 mg, 1.0 mmol, 2.0 equiv) and *N*,*N*-diisopropylethylamine (DIPEA, 359 μL, 4.0 equiv). The reaction mixture was stirred at room temperature for 3 h, then was added CH_3OH (500 μL) and stirred for another 30 min. The mixture solution was removed and washed with *N*,*N*-dimethylformamide (DMF, 20 mL × 3). The residue was added 20 mL 20% piperidine/DMF solution and stirred at room temperature for 30 min, then washed with DMF (20 mL × 5) to obtain compound **1**. Subsequently, *N*,*N'*-disuccinimidyl carbonate (DSC, 256 mg, 1.0 mmol, 2 equiv) and DIPEA (359 μL, 4.0 equiv) was added in 20 mL DMF and stirred at room temperature for 3 h. The mixture solution was removed and washed with DMF (20 mL × 3). The residue was added H-Glu(OtBu)-HCl (296 mg, 1.0 mmol, 2 equiv), DIPEA (359 μL, 4.0 equiv), and DMF (20 mL). The mixture was stirred at room temperature for 2 h, then removed the solution and washed with *N*,*N*-dimethylformamide (DMF, 20 mL × 3). The residue was added 20 mL 2% hydrazine monohydrate/DMF solution and stirred at room temperature for 30 min, then washed with DMF (20 mL × 5) to obtain compound **2**. Fmoc-D-2-Nal-OH(658 mg, 1.5 mmol, 3.0 equiv), HOBt (203 mg, 1.5 mmol, 3.0 equiv) and DIC (232 μL, 1.5 mmol, 3.0 equiv) were added, followed by 20 mL DMF, stirred at room temperature for 1.5 h, removed the DMF, washed with DMF (3 times), then 20 mL 20% piperidine/DMF was added, stirred at room temperature for 30 min, washed with DMF (5 times) to obtain the compound **3**. The residue was added Fmoc-Amstat-OH (569 mg, 1.5 mmol, 3.0 equiv), HOBt (203 mg, 1.5 mmol, 3.0 equiv), DIC (232 μL, 1.5 mmol, 3.0 equiv), and 20 mL DMF. The mixture was stirred at room temperature for 1.5 h, removed the DMF, washed with DMF (3 times), then was added 20 mL 20% piperidine/DMF, stirred at room temperature for 30 min, washed with DMF (5 times) to obtain the compound **4**. The residue was added Fmoc-Lys(Dde)-OH(799

mg, 1.5 mmol, 3.0 equiv), HOBt (203 mg, 1.5 mmol, 3.0 equiv), DIC(232 μL, 1.5 mmol, 3.0 equiv), and 20 mL DMF. The mixture was stirred at room temperature for 1.5 h, then was removed the DMF and washed with DMF (3 times). The residue was added 20 mL 20% piperidine/DMF and stirred at room temperature for 30 min, then was washed with DMF (5 times) to obtain the compound **5**. To the residue was added 1,4,7-triazacyclononane-1,4,7-triacetic acid (NOTA, 623 mg, 1.5 mmol, 3.0 equiv), HOBt (203 mg, 1.5 mmol, 3.0 equiv), DIC (232 μL, 1.5 mmol, 3.0 equiv), and DMF (20 mL) and was stirred at room temperature for 1.5h, removed the DMF, washed with DMF (3 times), then 20 mL 2% Hydrazine monohydrate/DMF was added, stirred at rt for 30 min, washed with DMF (5 times) to obtain the compound **6**. To the residue was added compound **7** (670 mg, 1.5 mmol, 3.0 equiv), HOBt (203 mg, 1.5 mmol, 3.0 equiv), DIC (232 μL, 1.5 mmol, 3.0 equiv), and DMF (20 mL), the mixture was stirred at room temperature for 1.5 h, then was removed the solution and washed the residue with DMF (3 times), DCM (2 times), and MeOH (2 times). After the residue was dried under high vacuum, to the residue was added 10 mL of mixture solution (95% TFA,2.5% TIS, 2.5% H_2O) and stirred at room temperature for 2.5 h. The mixture was flited and the filtrate was poured into 100 mL ice-ether to form large amount of solid. The crude product was dissolved in acetonitrile and water in a ratio of 1:1 (v/v) and purified via reverse-phase Zonran Bondysil C18 column (10 nm&10 μm, 30 × 250 mm, Zonran technologies company Ltd., Shanghai, China). A linear gradient ranging from 100% solvent A (0.1% TFA in water): 0% solvent B (0.1% TFA in MeCN) to 0% solvent A: 100% solvent B at 60 min was used at a flow rate of 20 mL/min. The product was collected and lyophilized to afford the title compound as white solid. MS (ESI-TOF) (m/z): calcd for $C_{72}H_{96}F_2N_{14}O_{19}$ ($[M + 2H]^{2+}/2$), 749.3, found, 749.9, (Fig. S1).

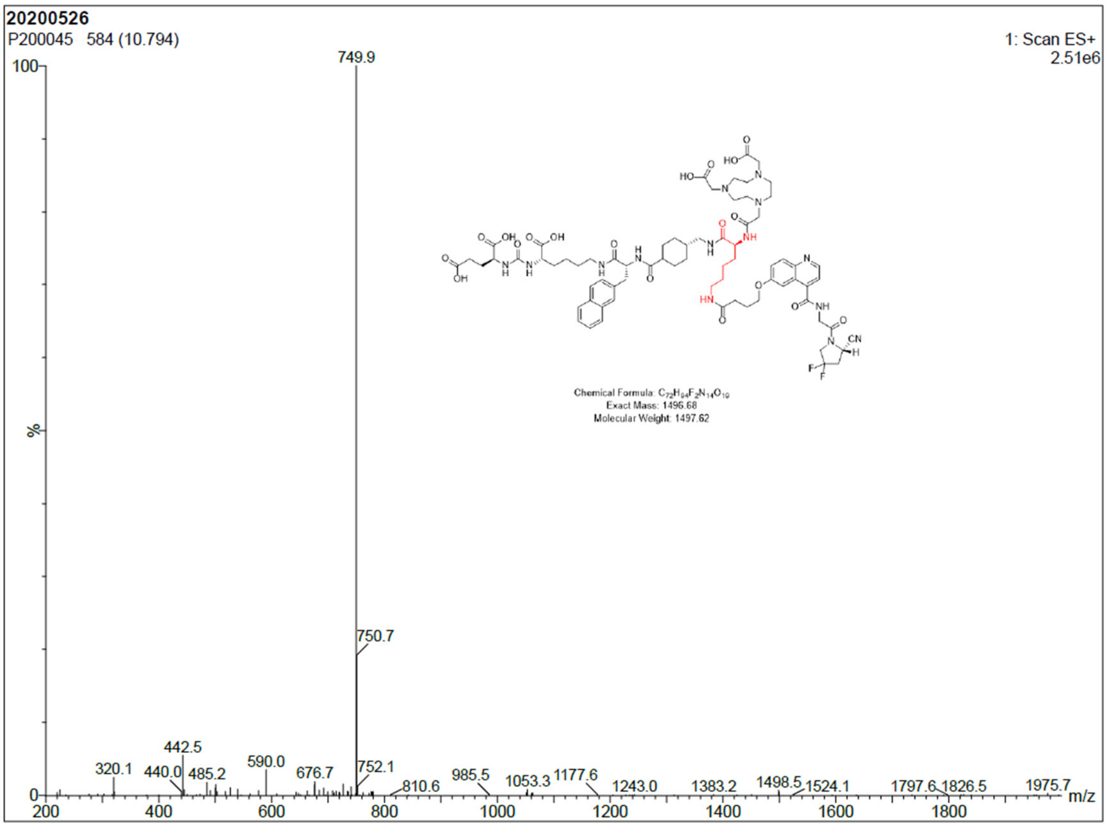

Figure S1. ESI-MS chromatogram of NOTA-PSMA-FAPI-01.

Synthesis of heterodimeric compound NOTA-PSMA-FAPI-02

Briefly, compound **6** (0.08 mmol, 1.0 equiv), compound **8** (50 mg, 0.08 mmol, 3.0 equiv), HOBt (10.9 mg, 0.08 mmol, 3.0 equiv) and DIC (12.3 µL, 0.08 mmol, 3.0 equiv) were added in 20 mL DMF, the mixture was stirred at room temperature for 1.5 h, then was removed the solution and washed the residue with DMF (3 times), DCM (2 times), and MeOH (2 times). After the residue was dried under high vacuum, to the residue was added 10 mL mixture solution (95% TFA,2.5% TIS, 2.5% H_2O) and stirred at room temperature for 2.5 h. Then the mixture was dried under high vacuum, then dissolved in 10 mL of mixture solution (95% TFA,2.5% TIS, 2.5% H_2O) and stirred at room temperature for 2.5 h. The mixture was flited and the filtrate was poured into 100 mL ice-ether to form large amount of solid. The crude product was dissolved in acetonitrile and water in a ratio of 1:1 (v/v) and purified via reverse-phase Zonran Bondysil C18 column (10 nm&10 µm, 30 × 250 mm, Zonran technologies company Ltd., Shanghai, China) A linear gradient ranging from 100% solvent A (0.1% TFA in water): 0% solvent

B (0.1% TFA in MeCN) to 0% solvent A: 100% solvent B at 60 min was used at a flow rate of 20 mL/min. The product was collected and lyophilized to afford the title compound as white solid. MS (ESI-TOF) (m/z): calcd for $C_{79}H_{108}F_2N_{16}O_{20}$ ([M + 2H]$^{2+}$/2), 819.4, found, 819.8, (Fig. S2).

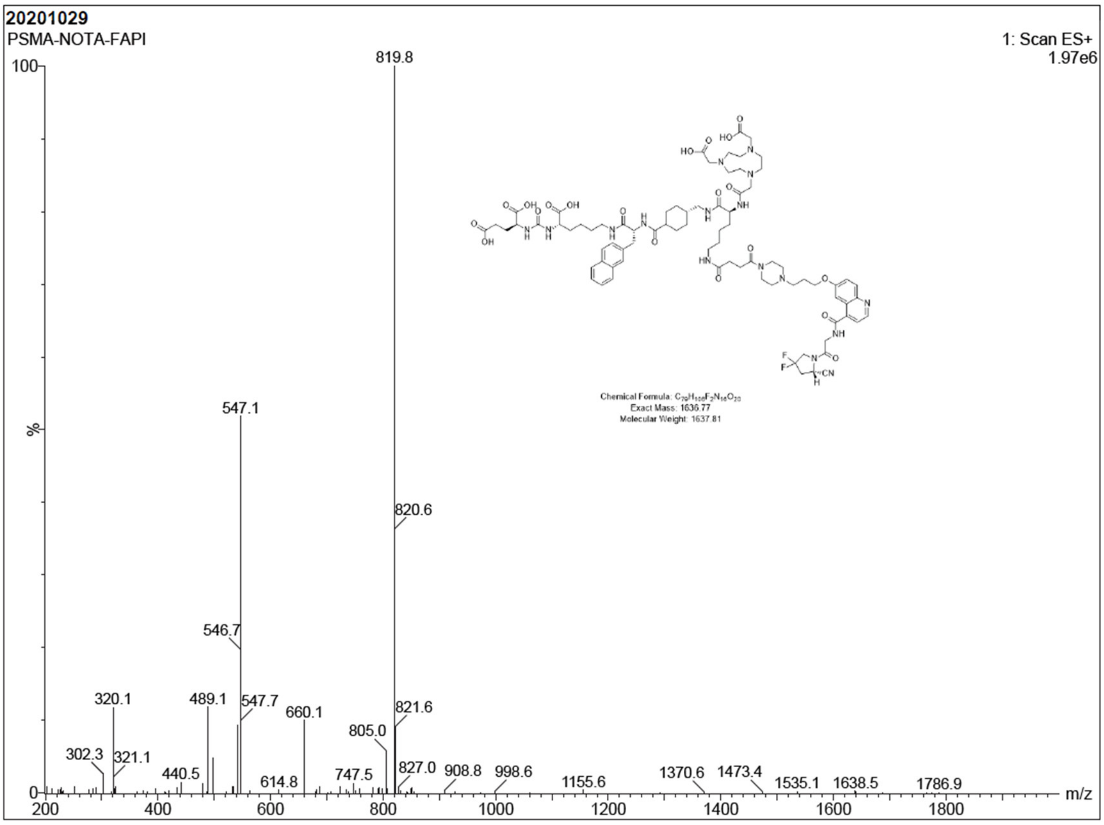

Figure S2. ESI-MS chromatogram of NOTA-PSMA-FAPI-02.

Synthesis of reference compounds [^{19}F]AlF-PSMA-FAPI-01/02

To a solution of NOTA-PSMA-FAPI-01 or NOTA-PSMA-FAPI-02 (0.30 μmol, 1.0 equiv) in 300 μL of 0.5 mol/L sodium acetate buffer (pH 4.0) was added $AlCl_3$ [15 μL, 0.30 μmol, 20.0 mmol/L, in sodium acetate buffer (0.2 mol/L, pH 4.0)], dimethyl sulfoxide (300 μL), and potassium fluoride aqueous (15 μL, 0.30 μmol, 20.0 mmol/L). The reaction mixture was heated at 100 ºC for 60 min. After cooling to room temperature, the reaction mixture was purified using reverse-phase XDB-C18 analytic column (ZORBAX Eclipse, 4.6 × 150 mm, 5 μm; Agilent Technologies, USA). A linear gradient ranging from 95% solvent A (0.1% TFA in water): 5% solvent B (0.1% TFA in MeCN) to 80% solvent A: 20% solvent B at 60 min was used at a flow rate of 1 mL/min

to afford the title compound. [19F]AlF-PSMA-FAPI-01, HRMS (ESI-TOF) (m/z): calcd for $C_{72}H_{90}AlF_3N_{14}O_{19}$ ($[M - 2H]^{2-}/2$), 769.3138, found, 769.3112 (Fig. S3). [19F]AlF-PSMA-FAPI-02, HRMS (ESI-TOF) (m/z): calcd for $C_{79}H_{102}AlF_3N_{16}O_{20}$ ($[M - 2H]^{2-}/2$), 839.3612, found, 839.3586 (Fig. S4).

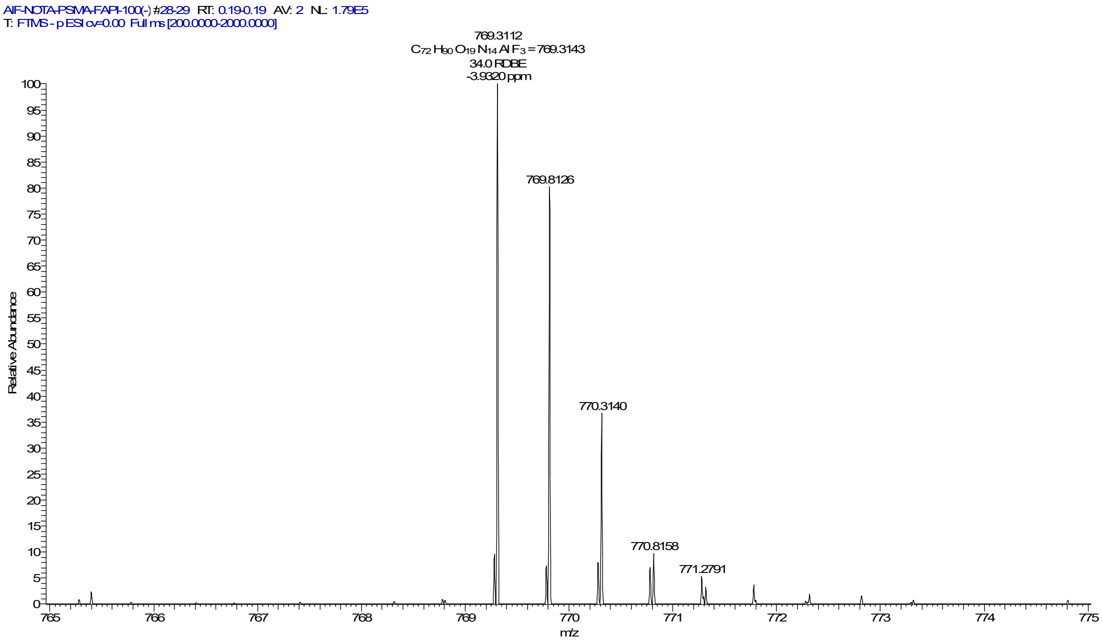

Figure S3. The High-resolution mass spectrometry for the reference compound [19F]AlF-PSMA-FAPI-01.

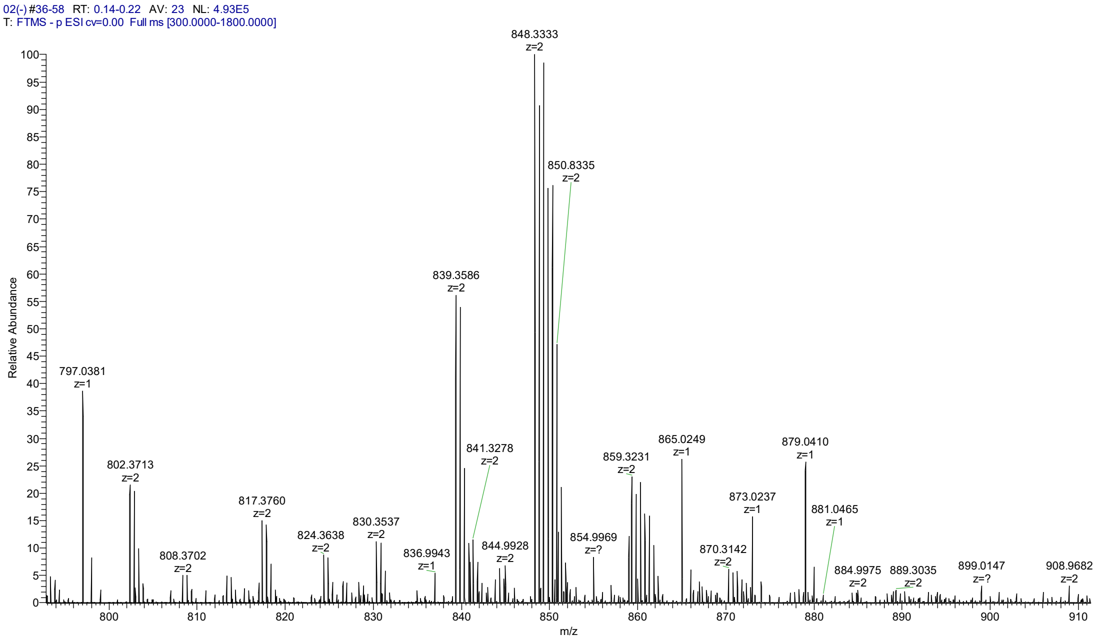

Figure S4. The High-resolution mass spectrometry for the reference compound [19F]AlF-PSMA-FAPI-02.

Molar activity

The molar activity of [^{18}F]AlF-PSMA-FAPI-02 was expressed as total radioactivity/molar ([^{19}F]AlF-PSMA-FAPI-02 + NOTA-PSMA-FAPI-02 + metal complexes of NOTA-PSMA-FAPI-02). We separately injected 20 μL of seven different concentrations (1.53, 0.765, 0.383, 0.192, 0.096, 0.048, 0.024 mmol/L of NOTA-PSMA-FAPI-02) into the HPLC and obtained the relevant UV area at 254 nm (Fig. S5). The concentration of total FAPI analogue ([^{19}F]AlF-PSMA-FAPI-02 + NOTA-PSMA-FAPI-02 + metal complexes of NOTA-PSMA-FAPI-02) in the [^{18}F]AlF-PSMA-FAPI-02 solution was calculated from the UV area from the retention time 8.8–9.6.

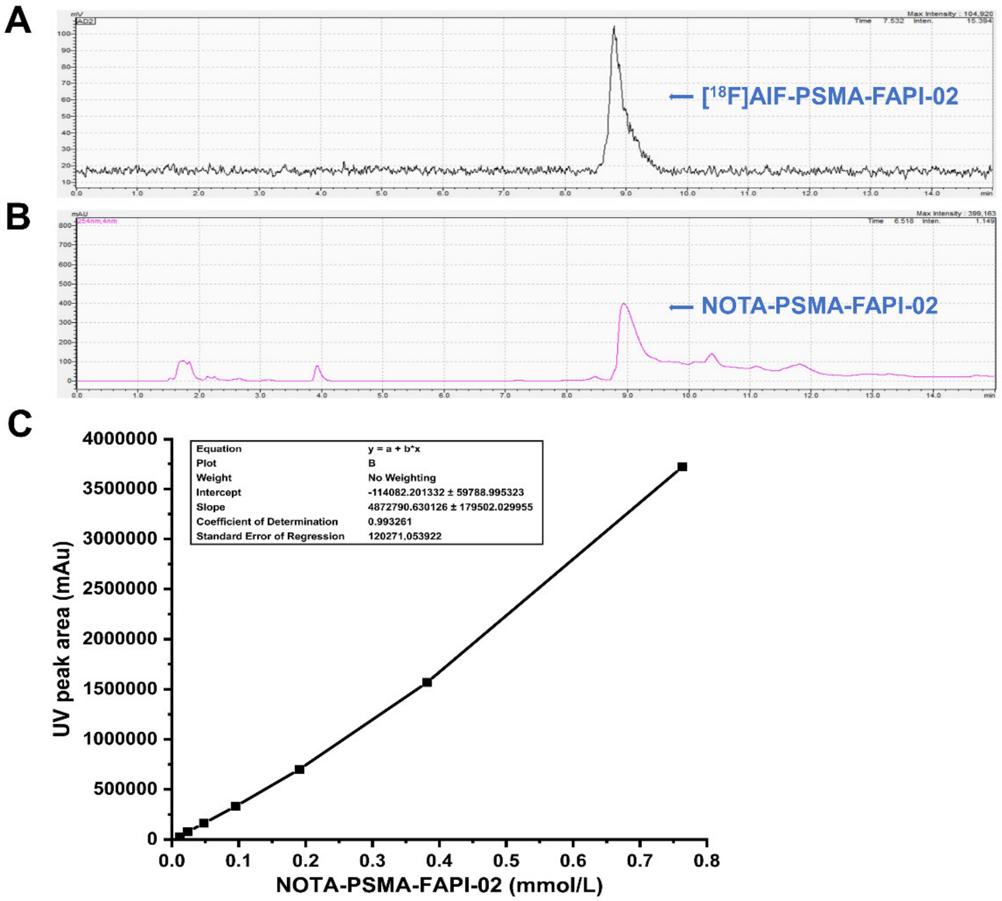

Figure S5. Representative HPLC chromatograms after injection of [^{18}F]AlF-PSMA-FAPI-02 solution, radiation detector (A); 254 nm UV detector (B); (C) Calibration curve of the HPLC UV detector for the quantification of the amount of NOTA-PSMA-FAPI-02 (linear regression of the amount of NOTA-PSMA-FAPI-02 vs. peak area (UV,

254 nm).

Cell uptake and blocking study

Table S1. Cell uptake and blocking study of [^{18}F]AlF-PSMA-FAPI-01, [^{18}F]AlF-PSMA-FAPI-02, and corresponding monomeric tracer [^{18}F]FAPI-42 or [^{18}F]AlF-PSMA-BCH in A549-FAP cells and 22Rv1 cells . Values are expressed as means ± SD (% ID/1 million cells).

		5min	15min	30min	60min	120min
A549-FAP	[^{18}F]AlF-PSMA-FAPI-01 Uptake *vs.* [^{18}F]AlF-PSMA-FAPI-01+DOTA-FAPI-04	8.15 ± 0.31 *vs.* 0.67 ± 0.07 $P < 0.01$	16.57 ± 0.98 *vs.* 0.71 ± 0.09 $P = 0.01$	23.73 ± 0.33 *vs.* 0.77 ± 0.01 $P < 0.01$	30.36 ± 3.53 *vs.* 0.83 ± 0.09 $P < 0.01$	28.23 ± 2.97 *vs.* 1.16 ± 0.06 $P < 0.01$
	[^{18}F]AlF-PSMA-FAPI-02 Uptake *vs.* [^{18}F]AlF-PSMA-FAPI-02+DOTA-FAPI-04	11.66 ± 0.39 *vs.* 1.28 ± 0.08 $P < 0.01$	17.03 ± 0.85 *vs.* 1.29 ± 0.38 $P < 0.01$	23.26 ± 1.59 *vs.* 1.31 ± 0.16 $P < 0.01$	27.02 ± 2.21 *vs.* 1.59 ± 0.65 $P < 0.01$	32.55 ± 1.42 *vs.* 1.71 ± 0.23 $P = 0.01$
	[^{18}F]FAPI-42 Uptake	11.63 ± 0.67	21.06 ± 1.41	26.85 ± 1.40	23.21 ± 1.81	18.46 ± 0.75
22Rv1	[^{18}F]AlF-PSMA-FAPI-01 Uptake *vs.* [^{18}F]AlF-PSMA-FAPI-01+2-PMPA	2.91 ± 0.17 *vs.* 1.14 ± 0.18 $P < 0.01$	3.41 ± 0.36 *vs.* 1.34 ± 0.15 $P = 0.004$	5.31 ± 0.21 *vs.* 1.41 ± 0.05 $P = 0.001$	8.53 ± 0.26 *vs.* 1.58 ± 0.09 $P < 0.01$	10.01 ± 0.21 *vs.* 1.73 ± 0.18 $P < 0.01$
	[^{18}F]AlF-PSMA-FAPI-02 Uptake *vs.* [^{18}F]AlF-PSMA-FAPI-02+2-PMPA	3.41 ± 0.51 *vs.* 0.21 ± 0.06 $P < 0.01$	6.34 ± 0.01 *vs.* 0.73 ± 0.07 $P < 0.01$	9.41 ± 0.14 *vs.* 0.76 ± 0.04 $P < 0.01$	10.68 ± 0.67 *vs.* 0.78 ± 0.09 $P < 0.01$	11.57 ± 0.64 *vs.* 0.86 ± 0.19 $P < 0.01$
	[^{18}F]AlF-PSMA-BCH	0.69 ± 0.11	1.40 ± 0.16	2.18 ± 0.11	5.88 ± 0.42	7.26 ± 0.67

Biodistribution and blocking study

Table S2. Biodistribution and blocking study results for the ^{18}F-labeled heterodimers [^{18}F]AlF-PSMA-FAPI-01 and [^{18}F]AlF-PSMA-FAPI-02 in A549-FAP or 22Rv1 tumor-bearing nude mice at 1 h p.i. and the comparison with the corresponding monomer [^{18}F]FAPI-42 or [^{18}F]AlF-PSMA-BCH. Values are expressed as the mean ± SD (%ID/g).

Organ	[^{18}F]AlF-PSMA-FAPI-01	Blocked	[^{18}F]AlF-PSMA-FAPI-02	Blocked	[^{18}F]FAPI-42	[^{18}F]AlF-PSMA-BCH*
Bone	4.09 ± 0.76	1.53 ± 0.21	5.53 ± 0.18	3.83 ± 0.02	7.29 ± 1.52	0.84 ± 0.39
Muscle	2.18 ± 0.48	0.32 ± 0.05	1.35 ± 0.36	0.85 ± 0.39	1.86 ± 0.32	0.31 ± 0.10
Lung	1.82 ± 0.18	1.66 ± 0.33	1.37 ± 0.22	0.90 ± 0.24	0.96 ± 0.11	0.86 ± 0.25
Brain	0.16 ± 0.03	0.09 ± 0.03	0.11 ± 0.03	0.07 ± 0.03	0.10 ± 0.01	0.14 ± 0.04
Heart	1.56 ± 0.14	0.41 ± 0.02	0.84 ± 0.17	0.28 ± 0.07	0.76 ± 0.09	0.45 ± 0.10
Liver	1.38 ± 0.11	0.79 ± 0.37	0.81 ± 0.22	0.41 ± 0.12	0.83 ± 0.19	0.47 ± 0.03
Kidney	23.88 ± 3.01	28.65 ± 6.05	23.10 ± 7.91	26.70 ± 10.80	1.86 ± 0.11	—
Kidney*	23.40 ± 9.22	2.85 ± 0.38	27.00 ± 11.90	2.94 ± 0.35	—	66.10 ± 18.80
Spleen	0.99 ± 0.17	0.67 ± 0.20	0.51 ± 0.16	0.30 ± 0.03	0.64 ± 0.04	1.33 ± 0.54
Gall bladder	2.02 ± 0.40	1.67 ± 0.32	2.23 ± 1.07	1.39 ± 0.20	10.58 ± 0.83	6.62 ± 4.25
Stomach	1.03 ± 0.13	0.91 ± 0.43	0.83 ± 0.42	0.37 ± 0.06	0.56 ± 0.03	0.41 ± 0.05
Small intestine	1.23 ± 0.21	1.04 ± 0.53	0.84 ± 0.33	0.55 ± 0.29	2.85 ± 0.91	0.78 ± 0.12
A549-FAP tumor	6.40 ± 0.32	1.38 ± 0.77	6.47 ± 0.76	1.11 ± 0.88	5.25 ± 0.27	—
22Rv1 tumor*	12.37 ± 0.40	5.03 ± 0.75	12.85 ± 2.43	6.85 ± 1.53	—	6.44 ± 1.78
Blood	2.36 ± 0.60	0.88 ± 0.62	1.98 ± 0.46	0.57 ± 0.19	1.52 ± 0.22	0.56 ± 0.19

Note: Organ distribution and blocking data are in A549-FAP tumor-bearing mice using DOTA-FAPI-04 inhibitor (3mg/kg), while labeled "*" means organ distribution and blocking data are in 22Rv1 tumor-bearing mice using 2-PMPA competitor (3mg/kg).
